# Temperature-dependent transport properties of CVD-fabricated n-GaN nanorods/p-Si heterojunction devices

**DOI:** 10.1039/d0ra05973k

**Published:** 2020-09-10

**Authors:** K. M. A. Saron, M. R. Hashim, M. Ibrahim, M. Yahyaoui, Nageh K. Allam

**Affiliations:** Physics Department, College of Science and Arts, Jouf University P. O. Box 756 Al-Gurayyat Saudi Arabia kamalmohammedabdalla@yahoo.com; Materials & Electronics Research Institute (MERI), The National Centre for Research Khartoum 2404 Sudan; Nano-Optoelectronics Research and Technology Laboratory, School of Physics University Sains Malaysia Penang 11800 Malaysia; Laboratoire de Physique des Matériaux: Structure et Propriétés, Faculté des Sciences de Bizerte, Université de Carthage 7021 Zarzouna Bizerte Tunisia; Energy Materials Laboratory, School of Sciences and Engineering, The American University in Cairo New Cairo 11835 Egypt nageh.allam@aucegypt.edu

## Abstract

We report on the structural, electrical, and transport properties of high quality CVD-fabricated n-GaN nanorods (NRs)/p-Si heterojunction diodes. The X-ray diffraction (XRD) studies reveal the growth of hexagonal wurtzite GaN structure. The current–voltage (*I*–*V*) characteristics of the n-GaN NRs/p-Si heterojunction were measured in the temperature range of 300–475 K. The ideality factor (*n*) and zero-bias barrier height (*ϕ*_B0_) are found to be strongly temperature-dependent. The calculated values of *ϕ*_B0_ are 0.95 and 0.99 eV according to Gaussian distributions (GD) and modified Richardson for GD, respectively, which are in good agreement with the band offset of GaN/Si (0.95 eV). A Richardson constant of 37 cm^−2^ K^−2^ was obtained from the modified Richardson plot, which is close to the theoretical value for p-Si (32 cm^−2^ K^−2^). The Gaussian distributions (GD) of inhomogeneous barrier height (BHs) and modified Richardson for GD of BHs with TE have also been used to explain the obtained transport properties.

## Abstract 2

We report on the structural, electrical, and transport properties of high quality CVD-fabricated n-GaN nanorods (NRs)/p-Si heterojunction diodes.

## Graphical abstract

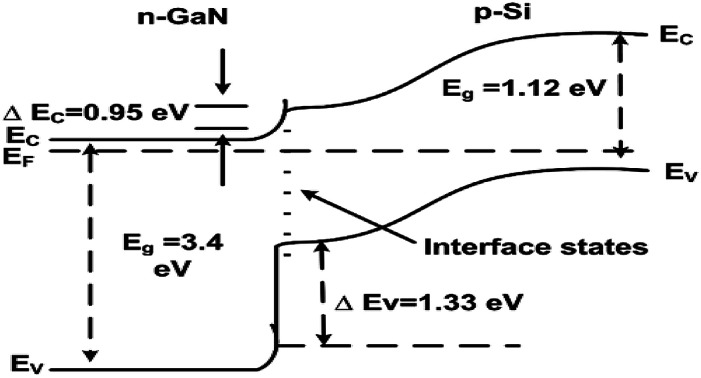

## 1. Introduction

Gallium nitride (GaN)-based-devices have drawn huge attention for future optoelectronic applications due to their unique properties, including high electron mobility, high thermal conductivity, and excellent thermal stability.^1,2^ The wide bandgap and high breakdown voltage of GaN are particularly crucial to ensure the functionality of the electronic devices at higher temperatures.^2,3^ Owing to the lack of native substrates, GaN-based devices are grown alternatively on various substrates such as Al_2_O_3_, SiC, and Si.^3–5^ Among those substrates, the growth of GaN on Si is of significant interest in terms of processing, thermal conductivity, integration, and low cost.^6^ The combined structures of GaN and Si on the same chip have been widely proposed for optoelectronic devices and p–n heterojunctions.^5–7^ One of the most significant challenges that limit their potential use is to obtain high-quality GaN structures on silicon substrates.^8–10^ To achieve high-quality GaN-based devices on Si, most of the researchers were mainly focusing on improving the growth techniques.^10,11^ Despite the significant efforts to improve the quality of GaN, the impact of structural defects on the electrical and optical properties remains a challenge.^12^ These defects in heterostructures arise from several factors such as atomic inhomogeneities at the interfaces, surface preparation, impurity concentration, and dislocations that lead to deterioration of electrical transmission across the device.^13–15^ The interface consists of high and low barrier areas, mainly as a result of the formation of different defects between semiconductors at the interface.^15–17^

Therefore, it is of great importance to understand the nature of electrical transport through GaN/Si heterojunctions. In order to improve the performance of devices, it is neccessary to study the electrical characteristics of GaN/Si Schottky interfaces at different temperatures. A rigorous analysis of the current–voltage (*I*–*V*) characteristics of the GaN/Si heterojunction diodes at different temperatures provides detailed information on the current flow through a single junction and the nature of the barrier formation at GaN/Si interfaces.^18–21^ However, there have been few previous studies on electrical transport through GaN/Si heterojunction diodes.^12,21,22^ For example, Bhat *et al.*^11^ studied the impact of nitridation on the electrical properties of the n-GaN/p-Si heterojunction. Xu and co-workers investigated the *I*–*V*–*T* characteristics of n-GaN/n-Si heterojunctions at different temperatures (18–400 K) and they reported an ideality factor of 10.^23^ Recently, Tuan *et al.*^24^ studied the electrical properties of p-GaN/n-Si at a testing temperature of (300–450 K) and they estimated a Schottky barrier height in the range 0.5 to 0.62 eV. However, to the best of our knowledge, there is no report on the *I*–*V*–*T* characteristics of n-GaN/p-Si diodes in the absence of buffer layers.

Herein, we demonstrate our attempt to grow GaN nanorods (NRs) directly on Si substrate and to examine the temperature-dependent electrical properties of the fabricated GaN NRs/p-Si heterojunctions. The obtained parameters, including ideality factor diode, barrier height (BHs), flat-band barrier height and Richardson constant, have been extracted from the thermionic emission and discussed.

## 2. Experimental details

GaN NRs have been grown by direct reaction of Ga with NH_3_ on Si substrate using chemical vapour deposition (CVD) system at 1050 °C for one hour. An alumina boat containing 0.2 g of Ga (99.99%) metal was placed at the center of a three-zone horizontal tube furnace (HTF) and Si (111) substrate was placed at 0.2 m away downstream from the Ga metal. The temperature of the center zone (Ga metal) was kept at 1000 °C during the evaporation process. The condensation products were deposited onto the Si substrate placed in a temperature zone of 1050 °C. An aqueous NH_3_ solution (120 mL) with a weight concentration of 25% solution was used as the nitrogen source and was kept at room temperature (RT). The N_2_ flow rate was maintained at 2 L min^−1^ under atmospheric furnace pressure during the growth process. The NH_3_ flow rate was controlled by the N_2_ flow-rate. When the temperature reached to growth temperature, NH_3_ gas was introduced into the HTF. After the reaction, the furnace was allowed to cool to RT naturally under an N_2_ flow rate of 2 L min^−1^. For further details, the reader may refer to (ref. 25). After the formation of GaN layer, the samples were cut into two pieces, and then the heterojunction GaN/Si diode was prepared at the nominal size of 1 cm^2^. The fabricated p–n heterojunction diodes were completed by depositing ohmic contact. The Ag–Al and Al ohmic contacts were deposited on the top of the GaN substrates and on the back of the Si substrates, respectively, through thermal evaporation. The metals were annealed at 450 °C using HTF in N_2_ flow for 10 minutes to realize optimal contact.

The morphological characterization of the sample was performed by field emission scanning electron microscopy (FE-SEM). The phase and purity of the GaN NRs were examined by X-ray diffraction (XRD) using Cu K_α1_ radiation (*λ* = 1.5406 Å) with a step size of 0.05°. The scanning range was between 2*θ* = 20° and 75°. The carrier type and concentration of the Si substrate were examined by Hall measurements using the HL5500PC system. After the contact formation, the sample was characterized by *I*–*V* in the dark condition. The electrical measurements were carried out by using a computer-controlled integrated source meter (Keithley 2400) at different temperatures (300–475 K). The operation temperature of the diode was measured using a calibrated K-type thermocouple mounted on the device.

## 3. Results and discussion

**Fig. 1:**
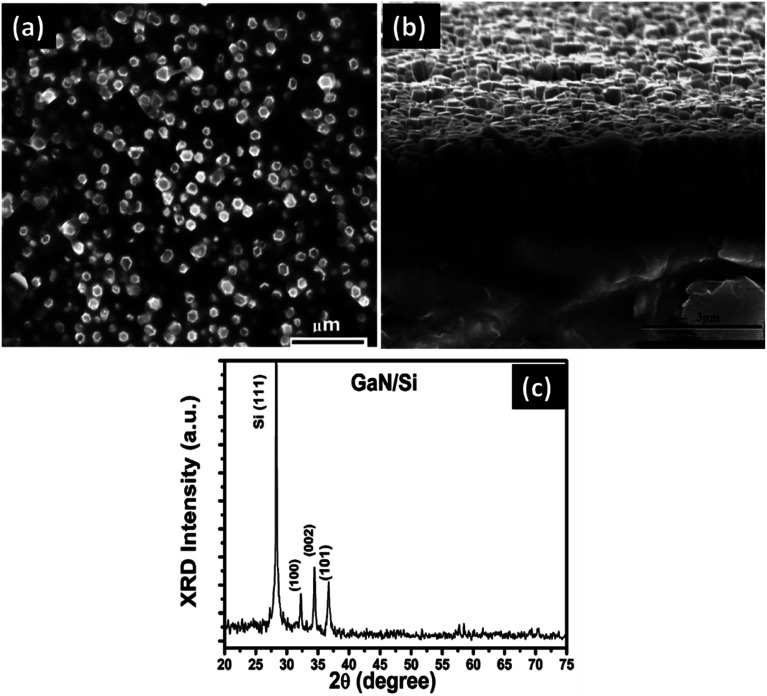
(a) top-view, (b) cross-sectional view of FESEM images, and (c) XRD pattern of the GaN NRs grown on Si substrate by CVD.

Fig. 1 shows the top and cross-sectional view of the FESEM images, and XRD pattern of the grown GaN on p-Si (111). High density nanorods (NRs) were covered the surface of the Si substrate as shown in Fig. 1a and b. The NRs facet was observed to attain a hexagonal shape with an average diameter in the range of 120 to 190 nm and a height less than 1 μm. The XRD spectrum, Fig. 1c, reveals three intense peaks corresponding to (100), (002), and (101) of GaN structures located at 32.3°, 34.6°, and 37.3°, respectively. These findings agree well with the reported standard values (JCPDS # 2-1078).^2,25^ The sharp and intense peaks indicate the good crystalline quality of the grown GaN NRs.

**Fig. 2:**
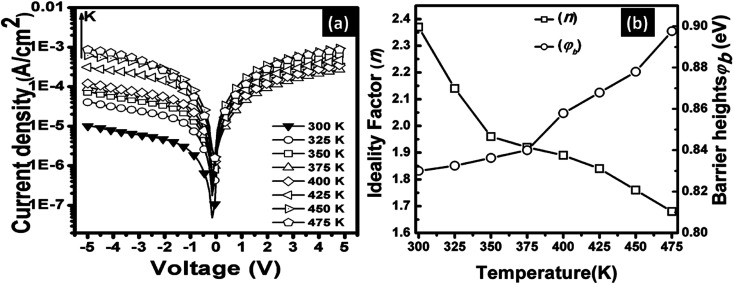
(a) Temperature-dependent *J*–*V* semi log plot for n-GaN/p-Si heterojunction and (b) the variations of ideality factor and barrier height as a function of temperature obtained from TE for GaN/Si heterojunction.

The electrical characteristics of the grown n-GaN NRs/p-Si heterojunction were monitored as a function of forward and reverse bias voltage conditions at 300–475 K. Fig. 2a shows *I*–*V* curves of the heterojunction device measured at different temperatures, indicating temperature-dependent behavior, which can be ascribed to the inhomogeneities at the interface.^16,18^ The current increases with increasing temperature due to the increase of the thermally-activated carriers and the reduction of the internal resistance. At high temperature, the rectifying nature deteriorates, possibly owing to the thermally-generated carrier tunneling.^17,18^ A slight decrease in the forward current at a higher temperature of 475 K was also observed. This behavior can be ascribed to the pronounced diffusion current at high bias voltage arising from the linear reduction in the barrier for electron and hole diffusion current. According to Einstein's relation, the diffusion current is strongly dependent on the carrier mobility.^18^ However, at higher temperatures, the phonon population increases resulting in decreased mobility. However, at a certain bias, an increase in temperature results in an increase in the forward current, an indicator that the current could be caused by the thermionic emission (TE). Thus, the current transport through a heterojunction at a forward bias can be understood based on the TE theory.^26^ The forward bias *I*–*V* measurements of the GaN/p-Si heterojunction can be given by eqn (1) and (2):^26,27^1
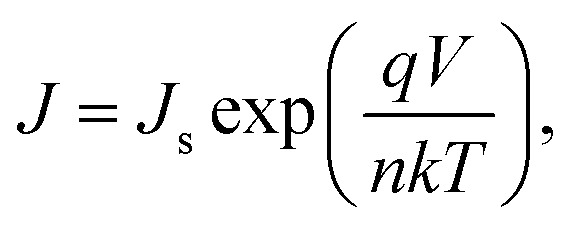
2
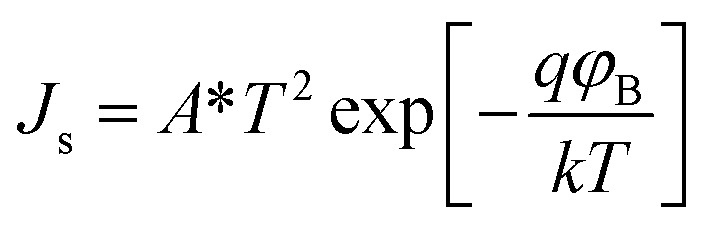
where *q* is the electronic charge, *V* is the applied forward bias voltage, *k* is the Boltzmann's constant, *T* is the absolute temperature, *n* is the diode ideality factor, *A* is the contact area, *J*_s_ is the reverse-bias saturation density current, *ϕ*_B0_ is the zero-bias barrier heights (BHs) of device, and A* is the effective Richardson constant (A* = 26.4 A cm^−2^ K^−2^ for n-GaN and 32 A cm^−2^ K^−2^ for p-Si).^16,28^ The values of BHs and the ideality factor (*n*) for the fabricated heterojunction were calculated as a function of temperature as extracted from the forward bias *I*–*V* curves. Both the saturation current density (*J*_s_) and *n* were estimated from the linear regions of the semi-log forward bias *I*–*V* curve according to eqn (3):^26^3
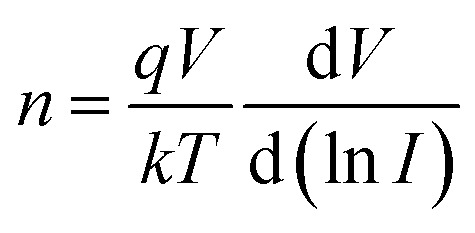
Upon plotting (ln *J*) against voltage, the slope gives the *n* and the intercept gives the *J*_s_. The obtained value of *J*_s_ is used to determine the zero bias BHs (*ϕ*_B0_):^26^4
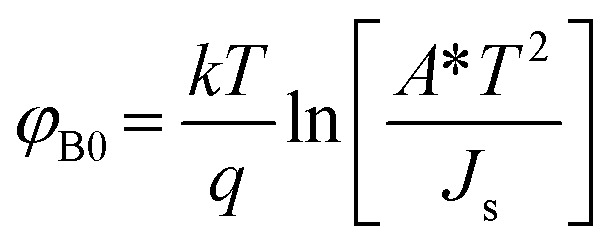


The zero bias BHs and *n* of the fabricated n-GaN/p-Si heterojunction diode were estimated at each temperature, as shown in Fig. 2b. It was observed that the BHs increase from 0.83 eV at 300 K to 0.90 eV at 475 K. As the temperature increases, more electrons would have sufficient energy to surmount the higher barrier. As a result, the dominant barrier height would increase with temperature.^29^ It was also observed that *n* deviates from unity and decreases with increasing temperature, which suggests the presence of an inhomogeneous barrier. The high values of *n* can be attributed to a wide distribution of low-Schottky barrier height (SBH) areas, tunneling through the interfacial layer, and a barrier inhomogeneities.^20^ Note that the dependence of *ϕ*_B_ and *n* on the temperature can be attributed to the heterogeneous and potential fluctuation at the interface.^16,18,30^ The BHs are possibly a function of the atomic inhomogeneities at the GaN/Si interfaces and the interface atomic structure, which are caused by nonuniformity of the interfacial charges, defects, and interfacial oxide layer composition.^16^ Consequently, the inhomogeneous BH may be varied as a result of the different types of defects that might exist at the GaN/Si interface. These observations are consistent with the TE theory and reported studies InGaN/Si barrier height^17,21^ and GaN/Si heterojunctions.^11,20,23^ However, in cases where the ideality factor is larger than unity, the TE would not be the only factor that promotes the transfer of current. As the BH depends on the applied voltage and, consequently, on the current flow across the interface, it is requisite to consider the standard field conditions. The electric field is zero across the interface under flat band (FB) conditions.

To extract more details on the barrier height, the flat band barrier height (FBBH) can be obtained from the *J*–*V* graphs, which is derived from the zero-bias barrier height (*ϕ*_B0_) *via*eqn (5) and (6):^26^

5 *ϕ*_BF_ = *nϕ*_B0_ − (*n* − 1)(*E*_F_ − *E*_V_)

**Fig. 3:**
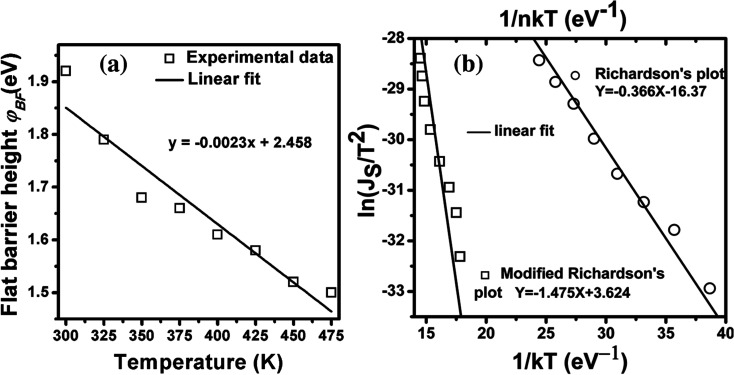
(a) Variation of flat band barrier heights as a function of temperature for n-GaN/p-Si heterojunction and (b) conventional Richardson plot, ln *I*_s_/*T*^2^*vs.* 1/*kT* and “modified” Richardson's plot ln *I*_s_/*T*^2^*vs.* 1/*nkT* for the fabricated heterojunction.

6
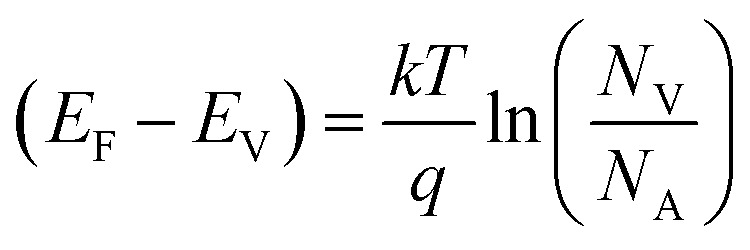
where *ϕ*_BF_ is the flat-band barrier height, *E*_F_ is the Fermi energy, *E*_V_ is the valence band energy, *N*_V_ the effective density of states of the valence band, and *N*_A_ is the carrier concentration. Hall measurements revealed that the carrier density of the Si substrate was ∼6 × 10^19^ cm^−3^ (p-Si). Notably, the deposition of the GaN layer formation causes the surface of the Si substrate to become heavily p-type doped through Ga in-diffusion.^5^ The GaN NRs layer is thought to have n-type behavior because of the *N* vacancies or/and oxygen contamination during the growth process.^5,31^ The FBBH was derived based on the assumption that the electron effective mass and *N*_A_ (the carrier concentration obtained from Hall measurement) do not vary with temperature. While the effective density of states in the valence band (*N*_V_) will change with the temperature and is given by eqn (7):7
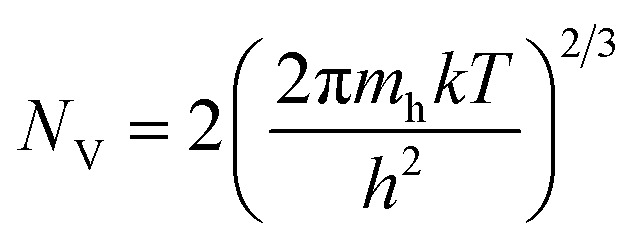
where *m*_h_ is the effective mass of hole (*m*_h_/*m*_o_ = 0.56 for p-Si), *T* is the absolute temperature, and *h* is the Planck's constant. The relation can be written as *N*_V_ (cm^−3^) = 4.3 × 10^14^(*T*)^3/2^ for p-type Si.^32^Fig. 3a shows the FBBHs as a function of temperatures. One can observe that the *ϕ*_BF_ slightly decreases with increasing temperature, whereas the zero-bias (ZB) BHs increase with increasing temperature. The difference between the ZB and the FBH values suggests low inhomogeneity and potential fluctuation at the interface.^18,19^ Futhermore, the temperature dependence of the *ϕ*_BF_ can be defined by eqn (8):

8 *ϕ*_BF_ = *ϕ*_BF_(*T* = 0) + *Tα*

where *ϕ*_BF_ (*T* = 0) is the barrier height extrapolated to zero-temperature, and *α* is the temperature cofficient of *ϕ*_BF_(*T*). The values of *ϕ*_BF_(*T* = 0) and *α* have been extracted fom the intercept and slope according to eqn (8). From the fit of the plot, the calculated values of *ϕ*_BF_(*T* = 0) and *α* were ∼2.5 eV and 0.0023 eV K^−1^, respectively. The value of *ϕ*_BF_(*T* = 0) is larger, which can be ascribed to the series resistance.^33^ Also, the value of average barrier height *φ̄*_B_ is lower than the *ϕ*_BF_(*T* = 0), and can be explained by lateral inhomogeneities of barrier heights.

Another method to extract the BH is through the application of a Richardson plot. From the experimental data measured in Fig. 2, the values of the saturation current (*J*_s_) were calculated at each temperature, and a “*conventional*” Richardson's plot, ln *I*_s_/*T*^2^*versus* 1/*kT*, is plotted as shown in Fig. 3b. From Richardson's plot, the barrier height and Richardson's constant were determined from the slope and intercept, respectively, using eqn (9):^26,27^9
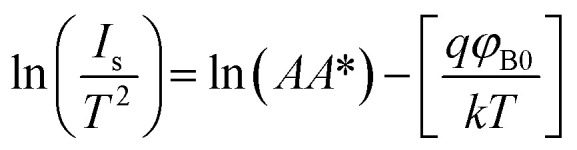


From this linear fit, the BH and the conventional Richardson's constant were 0.366 eV and 8.76 × 10^−8^ A cm^−2^ K^−2^, respectively. The obtained Richardson's constant is eight orders of magnitude smaller than the theoretical value of A* for p-Si.^32^ This deviation in A* is generally explained by the barrier inhomogeneity of the contacts, which means that it consists of high and low barrier areas at the interface.^34^ The inhomogeneous BH probably arises due to several types of defects present at the GaN NRs/Si hetero-interfaces.^35^ In addition, the effective area for current conduction (*A*_eff_) is significantly lower than the geometric contact area (*A*_geom_) due to the preferential current flow through the lower barrier height regions.^18^ In order to account for the temperature dependent *n* and BH, the modified Richardson's plot of ln(*I*_s_/*T*^2^) *versus* 1/*nkT* as proposed by Hackam and Harrop^36^ is shown in Fig. 3b. From the linear fit of the plot, the effective BH(*ϕ*_B0_) and modified Richardson's constant were determined from the slope and intercept, respectively. The values of effective *ϕ*_B0_ and the Richardson's constant A* were 1.475 eV and 37.48 A cm^−2^ K^−2^, respectively. The value of A* was eight orders of magnitude higher than that obtained from the conventional Richardson's plot. However, the value of A* obtained from the modified Richardson plot was found to be closer to the theoretical value for p-Si.^11,32^

The other method is to use the Gaussian statistics (GS) to modify the conventional Richardson plot to determine the BHs, taking into account SBH lowering due to an inhomogeneous contact.^37^ This method uses GS to relate experimental values of BH extracted from *I*–*V* measurements. Using eqn (1) for current over the barrier *ϕ*_B_ allows the values of BH calculated from the *I*–*V* data to be plotted against the inverse thermal energy to extractthe standard deviation (*σ*_s_) and mean BH *φ̄*_B_, which can be given by eqn (10):^26^10
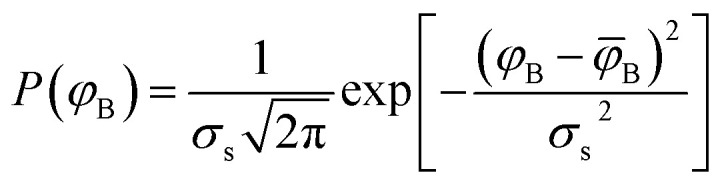
where 
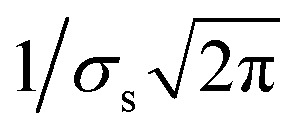
 is the normalization constant of the Gaussian barrier height distribution. The total current *I*(*V*) across a heterojunction diode containing barrier inhomogeneities is expressed by eqn (11):^37^11
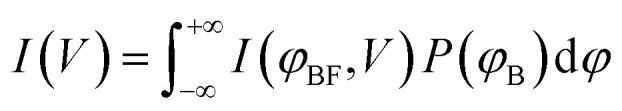
where *I*(*ϕ*_B_,*V*) is the current at a bias *V* for a barrier of height based on the ideal thermionic emission diffusion (TED) theory and *P*(*ϕ*_B_) is the normalized distribution function giving the probability of accuracy for barrier height. Solving the eqn (1) and (2), the total forward current can be given by eqn (12):^26,27,38^12
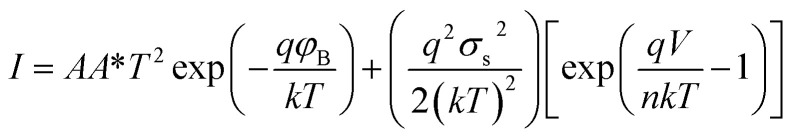


**Fig. 4:**
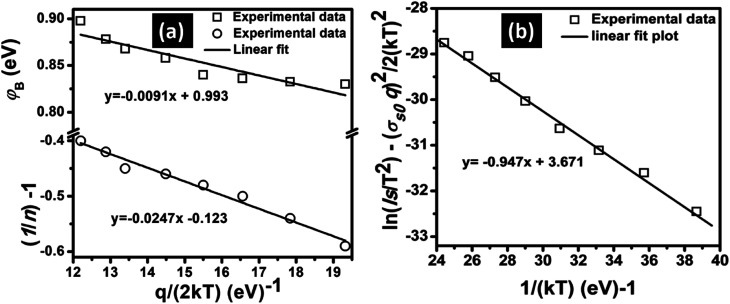
(a) The zero-bias apparent barrier heights extracted from *I*–*V* analysis plotted against the *q*/2*kT* and ideality factor *versus q*/2*kT* according to the GD of the BHs and (b) the modified Richardson plots ln(*I*_s_/*T*^2^) − 0.5*σ*^2^*q*^2^/(*kT*)^2^*vs.* 1/*kT* according to the GD of the BHs.

Applying the above integration (Eq.(11)), the current *I*(*V*) can be derived through a Schottky barrier at a forward bias but with a modified barrier as:^38^13
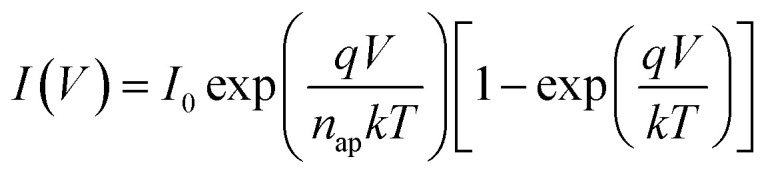
where *ϕ*_ap_ and *n*_ap_ are the apparent barrier height at zero bias and apparent ideality factor, respectively, and can be resolved by eqn (14) and (15):^37,38^14
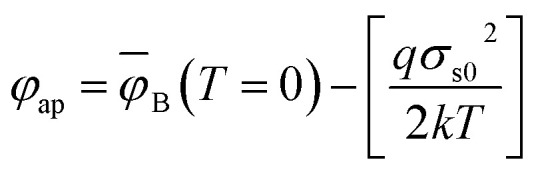
15
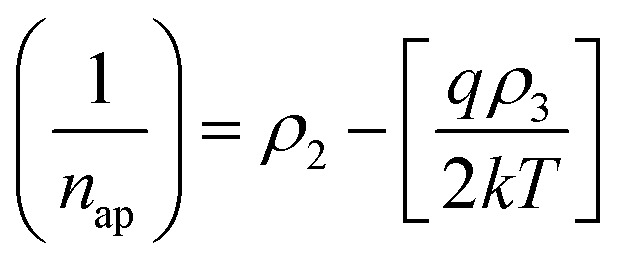
The mean BH (*φ̄*_B_) and standard deviation (*σ*_s_) are assumed to be linearly bias-dependent on Gaussian parameters. The values of *φ̄*_B_ and *σ*_s_ can be expressed as: *ϕ*_B_ = *ϕ*_B0_ (*T* = 0) + *ρ*_2_*V* and *σ*_s_ = *σ*_s0_ + *ρ*_3_*V*, where *ρ*_2_ and *ρ*_3_ are voltage coefficients, which may depend on temperature. The voltage coefficients quantify the voltage deformation of the barrier height distribution.^38^ The value of *σ*_s_ is usually very small therefore, can be ignored.^39^ Verification of *σ*_s0_ and *ϕ*_B0_ values can be carried out using a modified Richardson plot. Fig. 4a shows the zero-bias apparent barrier heights extracted from *I*–*V* analysis plotted against the *q*/2*kT* and ideality factor *versus q*/2*kT* according to the Gaussian distribution (GD) of the BHs. The plot of *ϕ*_B_*versus q*/2*kT* should be a straight line. The values of *σ*_s0_, and *φ̄*_B_ were extracted from the slope and intercept and found to be 0.0091 V and 0.993 eV, respectively. However, the voltage coefficients *ρ*_2_ and *ρ*_3_, which can be extracted from (1/*n*) − 1 *versus q*/2*kT*, were also reported in Fig. 4. From the intercept and slope of the linear fit, voltage coefficients *ρ*_2_ of 0.123 and *ρ*_3_ of 0.025 eV were obtained. The linear behavior plot shows that the ideality factor expresses the voltage deformation of the GD of the SB contact. The structure with the best rectifying performance presents the best barrier homogeneity with the lower value of the standard deviation.

Verification of *σ*_s0_ and *ϕ*_B0_ values can be carried out using a modified Richardson plot. The Richardson plot can be modified by combining eqn (2), (14) and (15) such that:16
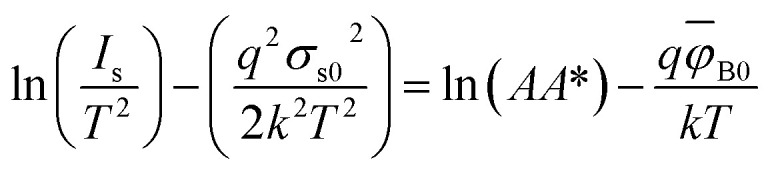


Fig. 4b shows the modified Richardson's plot of ln(*I*_s_/*T*^2^) − *σ*_s0_^2^*q*^2^/2(*kT*)^2^*versus* 1/*kT*. From the slope and the intercept of the modified plot, the mean *φ̄*_B0_ and modified Richardson's were extracted. The value of mean *φ̄*_B0_ obtained is 0.95 eV. Also, using eqn (2), the intercept (= ln AA*) at the ordinate determined A* for a given diode area (*i.e. A* ∼ 1 cm^2^) as 39.25 A cm^−2^ K^−2^. From the modified Richardson's plot and according to eqn (16), the obtained value of *ϕ*_B0_ = 0.95 eV is consistant with that of *ϕ*_B0_ = 0.99 eV extracted from the plot in Fig. 3a and lower than that of *ϕ*_B0_ = 1.475 eV from Fig. 3b. Furthermore, the barrier heights (0.99 and 0.95 eV) are closely similar to the conduction band offset of GaN/Si heterojunction (0.95 eV), which renounces the thermally generated carrier tunneling.^20^ The preceding results confirm that predominant current transport is not the only method for ascertaining barrier height inhomogeneities (BHI) in GaN/Si heterojunction sample. The results also establish that Gaussian distributions (GD) of BHs and modified Richardson for GD of BHs are also applicable. Table 1 summarizes the obtained values of BHs and Richardson's constant according to the modified Richardson's plot, GD of BHs, and modified GD of BHs methods. The values of BHs and Richardson constant, which were extracted using the modified Richardson's GD of BHs, are more acceptable. The outcome also concurs with the theoretical values.

**Table 1:** The values of BHs and Richardson constant of the fabricated GaN/Si heterojunction

Methode	BHs (eV)	Richardson constant (A cm^−2^ K^−2^)
Modified Richardson's plot	1.475	37.48
GD of BHs	0.99	—
Modified GD of BHs	0.95	39.25

**Fig. 5:**
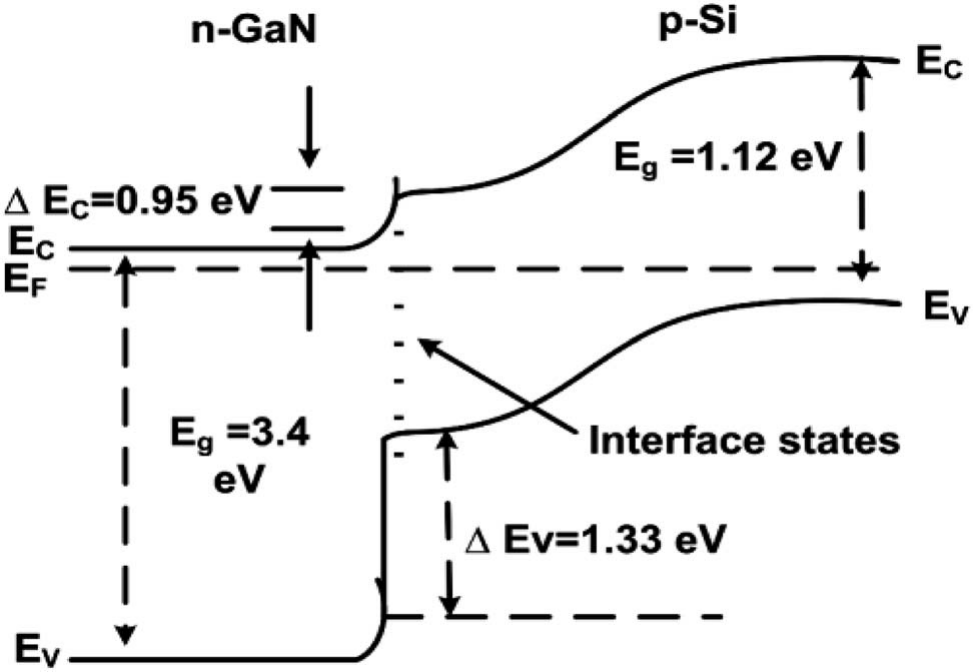
Schematic energy band alignment diagram of the n-GaN/p-Si heterojunction under thermal equilibrium.

Fig. 5 shows the possible band alignment diagram of n-GaN/p-Si heterojunction. The bandgaps (*E*_g_) and electron affinities (*χ*) used are *E*_g(Si)_ = 1.12 eV, *χ*_(Si)_ = 4.05 eV, *E*_g(GaN)_ = 3.4 eV, and *χ*_(GaN)_ = 3.1 eV, respectively, hence the conduction band offset (Δ*E*_C_ = *χ*_(GaN)_ − *χ*_(Si)_) is 0.95 eV, which is much smaller than the valence band offset, *i.e.*, Δ*E*_V_ = Δ*E*_g_ − Δ*E*_C_ = (1.33 eV).^35,39^

## 4. Conclusions

In conclusion, a high-quality n-GaN NRs/p-Si heterojunction device has been successfully fabricated. The relatively sharp diffraction peaks in the XRD spectra indicated the growth of highly crystalline GaN nanorods with wurtzite structure. The temperature dependence of the *I*–*V* characteristics of the fabricated heterojunction has been discussed. Using the TE model, the ideality factor (*n*) and barrier height (BH) of the heterojunction device were determined from the forward *I*–*V* curves under dark conditions. It is found that the values of ideality factor decreases with increasing the temperature, which suggests the presence of an inhomogeneous barrier. Deviations of *n* from unity reveal that TE will not be the only way that promotes the current transfer. It is observed that the *ϕ*_BF_ slightly decreases with increasing temperature, whereas the zero-bias (ZB) BHs increase with temperature. We have obtained a temperature coefficient BH of (*α* = 2.3 meV K^−1^) for this heterojunction. The measured value of the effective barrier height (∼1.48 eV) *via* the modified Richardson's plot is larger than that of BHs of 0.99 eV and 0.95 eV obtained by GD and modified Richardson GD, respectively. The calculated values of BHs and Richardson constant, which were extracted from the modified Richardson's GD of BHs, are reasonably acceptable.

## Data availability statement

The data that supports the findings of this study are available upon request.

## Conflicts of interest

There are no conflicts to declare.

## Supplementary Material
